# Sugarcane nitrogen nutrition estimation with digital images and machine learning methods

**DOI:** 10.1038/s41598-023-42190-2

**Published:** 2023-09-11

**Authors:** Hui You, Muchen Zhou, Junxiang Zhang, Wei Peng, Cuimin Sun

**Affiliations:** 1 College of Mechanics, Guangxi University, 100 East University Road, Nanning, 530004 Guangxi China; 2 Guangxi Vocational University of Agriculture, No. 249, East Daxue Road, Nanning City, 530007 Guangxi China; 3 College of Computer and Electronic Information, Guangxi University, 100 East University Road, Nanning, 530004 Guangxi China

**Keywords:** Electrical and electronic engineering, Mechanical engineering

## Abstract

The color and texture characteristics of crops can reflect their nitrogen (N) nutrient status and help optimize N fertilizer management. This study conducted a one-year field experiment to collect sugarcane leaf images at tillering and elongation stages using a commercial digital camera and extract leaf image color feature (CF) and texture feature (TF) parameters using digital image processing techniques. By analyzing the correlation between leaf N content and feature parameters, feature dimensionality reduction was performed using principal component analysis (PCA), and three regression methods (multiple linear regression; MLR, random forest regression; RF, stacking fusion model; SFM) were used to construct N content estimation models based on different image feature parameters. All models were built using five-fold cross-validation and grid search to verify the model performance and stability. The results showed that the models based on color-texture integrated principal component features (C-T-PCA) outperformed the single-feature models based on CF or TF. Among them, SFM had the highest accuracy for the validation dataset with the model coefficient of determination (R^2^) of 0.9264 for the tillering stage and 0.9111 for the elongation stage, with the maximum improvement of 9.85% and 8.91%, respectively, compared with the other tested models. In conclusion, the SFM framework based on C-T-PCA combines the advantages of multiple models to enhance the model performance while enhancing the anti-interference and generalization capabilities. Combining digital image processing techniques and machine learning facilitates fast and nondestructive estimation of crop N-substance nutrition.

## Introduction

Nitrogen (N) is essential for crop growth and development and is involved in many critical physiological processes. However, improper management of N fertilization may lead to agro-environmental pollution and reduce crop quality^1–3^. Non-destructive crop detection techniques, such as SPAD value detection and hyperspectral detection, have been widely used to diagnose the N-supplement nutritional status of crops^4–6^. However, these techniques usually require specialized equipment and technology and are costly and limited in use, not applicable to small-scale agricultural production.

Digital cameras, as a widely used tool for image acquisition, can assess crop growth and nitrogen nutritional status^7, 8^. The cost of using such equipment is more economical than other spectral instruments. In contrast, rich crop image information such as spectrum, texture, area, and relative space can be collected and thus computed as image characterization parameters. The correlation between these feature parameters and crop nutritional parameters is typically quantified by regression analysis^9–11^. Earlier studies have analyzed images through various color models to estimate leaves' N-vegetation content and chlorophyll concentration^12–14^. It is worth noting that image texture features have demonstrated a more extraordinary ability to estimate crop parameters than a single vegetation index. Thus, they have also been used for crop N content estimation^15, 16^ or combined with other parameters to enhance estimation accuracy^17^. Although good accuracy can be obtained using regression models for crop nutrient estimation in the same study^18^, the best estimation model varies for different crop varieties or nutrient parameters^19–21^. This suggests that simple regression models cannot generalize in estimating crop nutrition parameters.

Machine learning (ML) models, relying on autonomous programming and data processing, have the advantage of rapidly parsing large amounts of information and constructing models^22, 23^. Accompanied by advances in remote sensing technology, image sensing technology, and computer processing technology, ML methods have gradually gained popularity in the field of precision agriculture, covering a wide range of applications, such as agricultural product categorization, crop stress detection, pest and disease identification, and crop nutrition monitoring^24–27^. Standard algorithms for ML, such as support vector machine regression (SVR), artificial neural network (ANN), and random forest (RF) regression, exhibit excellent performance for accurate identification, classification, quantification, and prediction of a large number of feature parameters in crop images^28, 29^. However, the single ML model's anti-interference ability and learning efficiency are lacking. In contrast, Stacking model fusion, a multilayer model integration framework, combines the advantages of multiple models, such as SVR and ANN, to effectively improve model performance and generalization ability^30, 31^. Combining digital cameras and machine learning models provides an innovative tool for precision agriculture to predict crop nutritional status at a low cost accurately. Accurate forecast of crop nutritional status at low cost. This technology provides guidance to understand crops and make informed decisions sincerely but also reduces manual labor, improves agricultural management efficiency, and achieves sustainable agriculture's environmental goals^32, 33^. With the increased ease of access to and management of crop monitoring data and the expansion of data volume, using complex models to establish relationships between input variables and crop N-vegetable nutrition parameters has become a trend^34–36^.

Although ML models have demonstrated higher accuracy than simple regression models in crop nutrition diagnosis^19, 37, 38^, most of the studies have focused only on color features as input variables^39–41^ and have not adequately investigated the effects of other types of feature parameters on the accuracy of the models, and at the same time, the generalization performance of fusion models compared to other ML models is not yet clear. This study aimed to evaluate the accuracy and generalization performance of the regression methods under different input variables, intending to determine the optimal model for sugarcane N nutrition estimation from digital camera images. For this purpose, we selected leaves at tillering and elongation stages, which are the most efficient for N fertilization management in sugarcane. We captured their images with the help of a commercial digital camera under natural light conditions. After de-illumination and de-backgrounding, the color and texture parameters were extracted, and further correlation and principal component analyses were performed to serve as inputs for the three methods of evaluating sugarcane N-nutritional parameters, namely, multivariate linear regression (MLR), random forest regression (RF), and Stacking fusion model (SFM)Variables.

## Methods

### Experiments description

Field trials were conducted in the subtropical agricultural science and new city of Chongzuo City, Guangxi Autonomous Region, China (107.8°E, 22.5°N). The area belongs to the subtropical monsoon climate zone, with an average annual temperature of 22.9 °C, a total annual sunshine duration of 1731.2 h, and a total annual rainfall of 1182.5 mm. The primary chemical properties of the soil layer are as follows: total nitrogen (N) content, 0.97 g/kg; total phosphorus (P) 63.92 mg/kg; total potassium (K) 102.72 mg/kg; soil organic carbon, 11.46 g/kg; pH 5.12.

The experiment was carried out in 2022–2023 in the main production area of sugarcane, and the variety of sugarcane was ROC22 (New Taiwan Sugar 22). Fertilizers were slow-release fertilizers for sugarcane, and a single application method was used, i.e., 100% of the annual planned fertilizer application was applied as a base fertilizer at the beginning of sugarcane planting. The site manager authorized all collection activities during the trial, and the relevant Chinese laws and regulations were strictly adhered to to ensure sustainable sample collection. Only one leaf was collected from each sugarcane stem to minimize potential disturbance to the plant and the farmland.

### Sample collection and N content determination

Sixty plants of different lengths were randomly selected from each sugarcane tillering stage (2022.7.28-30) and elongation stage (2022.9.20-22), respectively, and each plant was collected from the top end of the morphology downward, the 1st fully expanded leaf. The collected samples were washed with 1 + 19 hydrochloric acid solution for 2 min, washed twice with ultra-pure water, placed in an oven at 105 °C for 30 min to kill, and then adjusted to 65 °C to dry to constant weight. The dried samples were ground to powder, weighed 0.1 g in a decoction tube, decocted with H2SO4-H202, fixed to 150 ml, and the N content of cane leaves was determined using a continuous flow analyzer AA3_HR and calculated by Eq. (1).

1 $$N=\frac{\mathrm{m}\cdot \mathrm{V}}{\mathrm{M}}\times {10}^{-3}$$

where m is the instrument reading, mg/L; V is the volume of the fixed volume, ml; M is the sample mass, g.

While collecting samples, images of sugarcane leaves were taken using a Nikon SLR digital camera (Z5, Nikon Corporation, Japan). The camera was fixed at 0.3 m directly above the A3 whiteboard as the background for photographs. Images were taken under normal light. The camera resolution was fixed at 4016*6016 kept constant, and all images were saved in JPEG format.

### Image feature parameter extraction

In this paper, we propose an automatic image processing method that can effectively preprocess leaf images and extract their color features (CF) and texture features (TF). The method consists of two main parts: image pre-processing and image feature computation extraction. Among them, image pre-processing mainly includes de-illumination processing and leaf theme extraction.

#### Image pre-processing

The leaf images collected in the field environment have the effects of uneven light range and different light intensities, which significantly impact the image CF extraction results. Processing images using Multi-Scale Retinex with Color Restoration algorithm (MSRCR) based on Retinex color constancy theory can eliminate the effects of uneven illumination. The basic idea of Retinex theory is to preserve the essential reflective properties of objects by removing or reducing the effects of incident components in the original images^42–44^. The MSRCR algorithm adds a color restoration factor to MSR to adjust the defects of color distortion due to contrast enhancement in local areas of the image. It eliminates color bias^45–47^.

**Figure 1:**
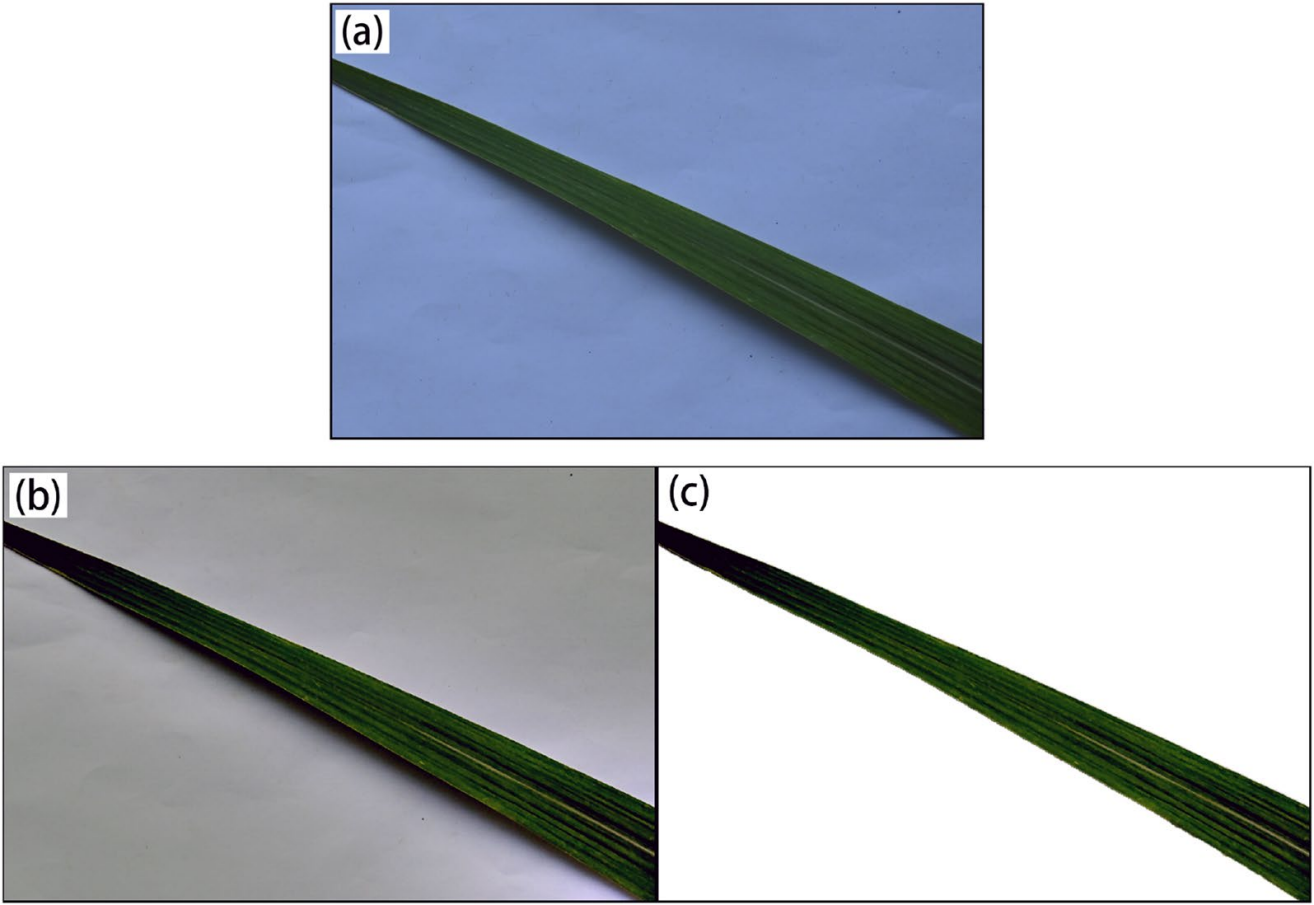
Pre-processed images of sugarcane leaves, where (**a**) denotes the original image, (**b**) denotes the de-illuminated image, and (**c**) denotes the de-contextualized image.

After the de-illumination process, to reduce the image redundancy and improve the accuracy of feature extraction, the U-Net deep network structure is used for Salient Object Detection (SOD) to extract the leaf body. U-Net is a deep network designed for image segmentation tasks, and its unique "U"-shaped structure (including two parts: encoder and decoder) and jump connection design make it perform well in object detection and image segmentation tasks. U-Net is a deep network designed for image segmentation tasks. Its special "U"-shaped structure (including two parts: encoder and decoder) and jump connection design make it perform well in object detection and image segmentation tasks. The encoder part downsamples the input image and extracts its deep features. In contrast, the decoder part reconstructs these features and up-samples them to segment the original image accurately. The U-Net deep network structure can maintain the high-resolution feature information of the picture and perform foreground and background segmentation with low computational costs ^48–50^. As shown in Fig. 1, the effect of the leaf body extracted by applying the MSRCR algorithm to eliminate the effect of illumination and using the U-Net deep network structure is remarkable.

#### Feature extraction

This study extracts CF parameters of 3 color models for analysis and modelling. The extraction of 3-channel mean values of images based on the RGB color model is performed first. Then other color features are calculated by algebraic operations and color space conversion, which are 24. The specific parameters are shown in Table 1.

**Table 1:** Selected color characteristics.

Color space	Percentage	Color features
RGB	50%	R,G,B,G/R,G/B,B/R, G-R,G-B,B-R,(B-G-R)/(B + G), (B-G-R)/(R + B),(B-G-R)/(G + R)
Normolized RGB	25%	r,g,b,gr,gb,br
HSV	12.5%	H,S,V
Lab	12.5%	L,a,b

TF is one of the main features to describe a leaf's condition visually and can be applied in conjunction with color features for leaf analysis^51, 52^. The Grey Level Co-occurrence Matrix (GLCM) algorithm was used to extract texture features. The probabilities of inter-pixel position relationships were calculated at the basic azimuth angles 0°, 45°, 90°, and 135° to obtain second-order statistics reflecting the characteristics of the target object, and the results were averaged over the four directions. To train the model, we selected five standard texture feature parameters, namely, Angular Second Moment (ASM), Inverse Difference Moment (IDM), contrast (CON), entropy (ENT), and Correlation (COR). The calculations for these parameters are as follows:

ASM is calculated using formula (1):

1 $$\begin{array}{c}ASM={\sum }_{i=0}^{L-1} {\sum }_{j=0}^{L-1}{p}^{2}\left(\mathrm{x},\mathrm{y}\right)\end{array}$$

IDM is calculated using formula (2):

2 $$\begin{array}{c}IDM={\sum }_{i=0}^{L-1} {\sum }_{j=0}^{L-1}\frac{p\left(x,y\right)}{1+{\left(1-j\right)}^{2}}\end{array}$$

CON is calculated using formula (3):

3 $$\begin{array}{c}CON={\sum }_{i=0}^{L-1} {\sum }_{j=0}^{L-1}{{\left|i-j\right|}^{2}p}^{ }\left(\mathrm{x},\mathrm{y}\right)\end{array}$$

ENT is calculated using formula (4):

4 $$\begin{array}{c}ENT={\sum }_{i=0}^{L-1}q {\sum }_{j=0}^{L-1}P\left(x,y\right)\mathrm{lg}p\left(x,y\right)\end{array}$$

COR is calculated using formula (5):

5 $$\begin{array}{c}COR=\frac{{\sum }_{i=0}^{L-1}ij {\sum }_{j=0}^{L-1}P\left(x,y\right)-{{\mathrm{u}}_{{1}^{ }}\mathrm{u}}_{{2}^{ }}}{{\partial }_{1}^{2}{\partial }_{2}^{2}}\end{array}$$

These parameters are widely used in texture analysis, and their formulas are well established in the literature.

### Estimation of N nutrient parameters

To estimate the N-nutrient parameters of sugarcane, three different regression methods were used in this study: backpropagation neural network (BPNN), random forest (RF) regression, and stacking fusion model regression (SFM). The five-fold cross-validation^53^ method was used in the modelling process, i.e., the original data were divided into five parts without repetition, one part was selected as the test set each time, and the remaining four parts were used as the training set for model training, and the data set division and modelling were repeated five times. Cross-validation can learn samples from multiple directions, which can effectively avoid falling into local minima and avoid overfitting problems to some extent. The mean values of root mean square error (RMSE), mean absolute percentage error (MAPE), and coefficient of determination (R^2^) is used to evaluate the model performance.

Before modelling construction, Pearson correlation analysis and principal component analysis were performed on N-nutrient parameters and sugarcane leaf image feature parameters. Different regression models were constructed using high correlation and principal component feature parameters.

#### Backpropagation neural network (BPNN)

In this study, the BPNN model was trained for the estimation of N-substance nutrient parameters of sugarcane. A five-fold cross-validation method was used to train and validate the feature parameter data for BPNN. To determine the best hyperparameters, a cyclic traversal through all candidate parameters was performed using grid search to find the best-performing parameter combination within the specified parameter range. The performance of each BPNN model was tested for the number of hidden layers and the number of neurons per hidden layer from 1 to 30 and 1 to 100, respectively. Also, four activation functions, log-sigmoid, ReLU, Identity, and Tanh, were tested. Three weight optimizers, sgd, adam, and lbfgs, were used to calculate and validate the errors and minimize the loss of the network by modifying the weights and learning rates appropriately. The maximum training epoch was set to 15,000 with a regularization parameter of 1 × 10–4. Before training, all input variables and targets were normalized to a range of ± 1. During model training, the model error is calculated for the validation dataset each time the neural network parameters are updated.

#### Random forest (RF) regression

RF regression is a supervised machine learning algorithm based on integration learning. Its basic idea is to create homogeneous subsets by bootstrap aggregating and then grow a decision tree in each subset (number of trees: ntree). The prediction result of the RF regression model is the average of the output of all decision trees. Compared to other models, RF is insensitive to noise in the training set and considers only some input variables to split the tree nodes, which can effectively prevent autocorrelation between independent variables from affecting the results and avoid overfitting^54, 55^.

In this study, the feature parameters selected by data analysis were used as input variables for RF regression. The optimal parameters were found within the specified parameter range by grid search. The optimal tree trees (n_estimators) were tested for values between 10 and 100 in increments of 1. The minimum number of samples required to split the internal nodes (min_samples_split) and the minimum number of samples required at the leaf nodes (min_samples_leaf) were also tested for values between 1 and 5. Other than that, all other hyperparameters in the RF regression are set to default values and implemented using the RandomForestRegressor function in the scikit-learn library.

#### Stacking model fusion (SFM)

The Stacking algorithm is a multi-layer model integration framework. Its first layer consists of multiple base learners input to the original dataset for training and output. The output of each base learner is stacked in columns to form (m, p)-dimensional new data (m represents the number of samples, and p represents the number of base learners). Then the new data is submitted to the next layer of models for fitting. Compared with a single model, the stacking framework combines the advantages of multiple models with enhanced resistance to interference and generalization. All base learners use the five-fold cross-validation method, effectively preventing model overfitting^30, 31, 38^.

A multi-model fusion model for N-substance nutrient content prediction of sugarcane was developed under the Stacking integration framework, considering the data observation space of multiple models. Three base learners were set in the first layer of the framework, using Partial Least Squares Regression (PLS), Support Vector Regression (SVR) and BPNN as the base models, and the GridSearchCV algorithm determined the optimal hyperparameters. Among them, the number of principal components (ncomponent) retained by PLS is tested between 1 and 10; the penalty factor (C) of SVR takes the value of 1, the kernel function (kernel) is chosen between linear, poly, rbf and sigmoid, and the kernel coefficient (gamma) is tested between scale and auto; the optimal parameters of BPNN are were chosen as described before. The second layer model is set as a BPNN model and trained using the first layer's output data. An N content stacking fusion prediction model is finally obtained. The output data of each learner in the framework will be saved and analyzed.

### Data analysis

In this study, Pearson correlation coefficient (R^2^), root mean square error (RMSE), and mean absolute percentage error (MAPE) were used as evaluation metrics for the performance of multiple supervised machine learning models. Among them, the correlation coefficient R^2^ describes the strength of the linear relationship between variables and affects the explanatory power of the model; RMSE measures the size of the prediction error and reflects the model accuracy; and MAPE considers the relative degree of the error and assesses the acceptability of the model. The formulas are as follows:

6 $$\begin{array}{c}{R}_{ }^{2}=1-\frac{{\sum }_{i=1}^{n}{\left({y}_{i}-{\widehat{y}}_{i}\right)}^{2} }{{\sum }_{i=1}^{n}{\left({y}_{i}-{\overline{y} }_{ }\right)}^{2} }\end{array}$$

7 $$\begin{array}{c}RMSE=\sqrt[ ]{ \frac{1}{\mathrm{n}} {\sum }_{i=1}^{n}{{\left({\widehat{y}}_{i}-{y}_{i}\right)}^{2}}^{ }}\end{array}$$

8 $$\begin{array}{c}MAPE=\frac{1}{\mathrm{n}}{\sum }_{i=1}^{n}{{\left|\frac{{\widehat{y}}_{i}-{y}_{i}}{{y}_{i}}\right|}^{ }}^{ }\end{array}$$

The images were preprocessed, feature parameters calculated, statistical data tests, RF and Stacking models were constructed using Python 3.8 and Scikit-learn library v1.0.2. The IBM SPSS 27 software was used for the significance of differences and correlation analysis. All images were produced using Python 3.8 and Origin 2022.

## Results

### Correlation analysis of N content and image feature parameters (CA)

**Figure 2:**
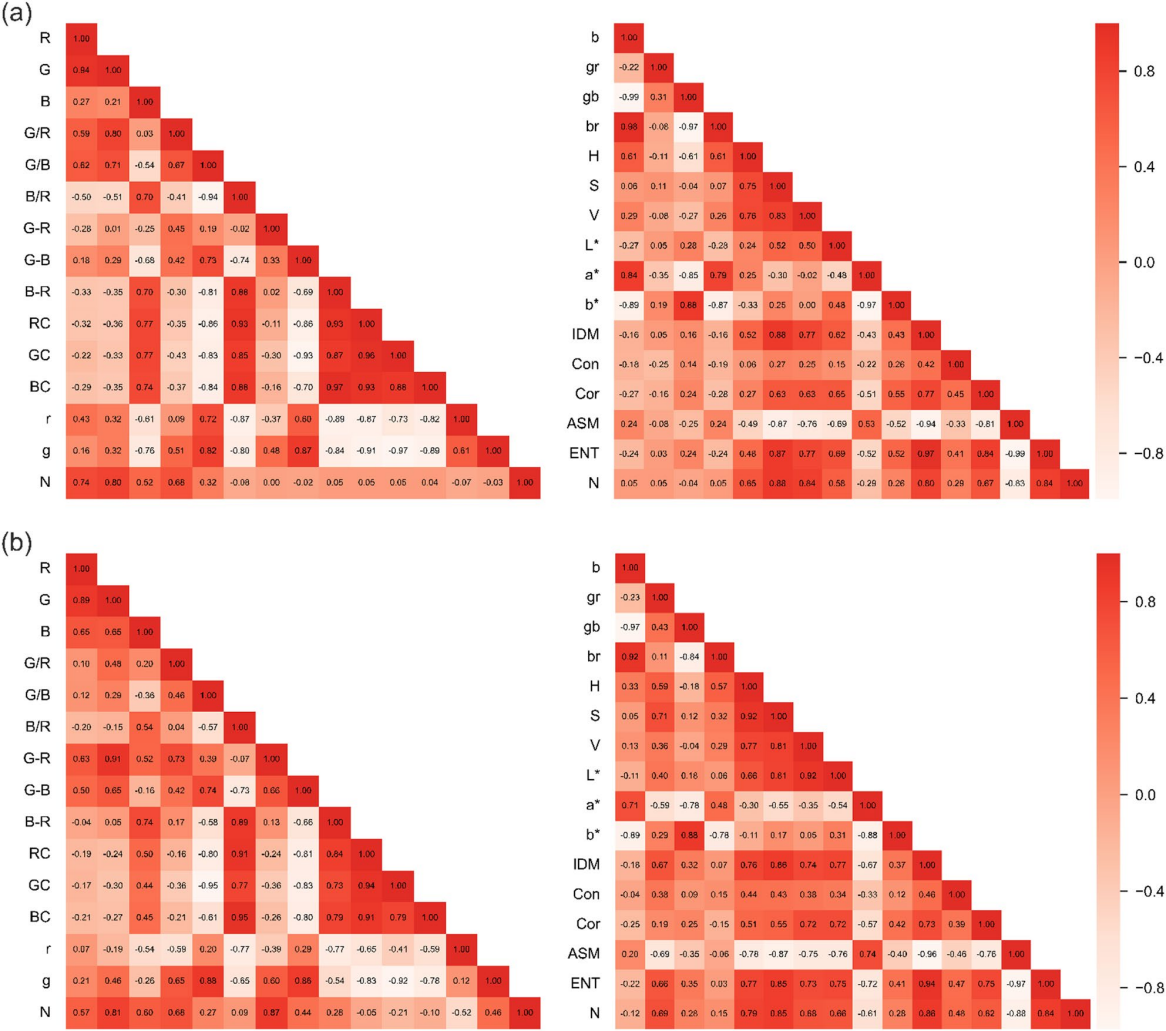
Heat map of correlation between N content of sugarcane leaves and image characterization parameters. Where (**a**) is the heat map at the tillering stage and (**b**) is the heat map at the elongation stage.

For different periods of cane leaves, this study conducted CA on the image features of leaf N content in each period and demonstrated the correlation by correlation heatmap (see Fig. 2). The analysis results showed that in the tillering period, eight color parameters and three texture parameters exhibited strong correlations, among which seven parameters, including R, G, S, V, IDM, ASM, and ENT, had the best correlation with N content, with correlation coefficients greater than 0.7 (ranging from 0.74 to 0.88). As for the elongation period, 14 color parameters and three texture parameters reflected strong correlations, among which G, (G-R), H, S, IDM, ASM, and ENT had the best correlations, with correlation coefficients greater than 0.7 (ranging from 0.79 to 0.88).

### Principal component analysis (PCA)

In this study, the 24 selected color features are obtained by algebraic operations and color space transformations of the three basic RGB color features, and there is a strong correlation between these features, which increases the complexity of the problem analysis. To reduce the number of features that need to be analyzed and, at the same time, minimize the loss of information contained in the original parts, the elements were downscaled, and PCA was used to downscale the color and texture features. After calculation, the variance contribution ratio of each principal component (PC) was obtained, as shown in Table 2. According to the Kaiser-Harris criterion, PCs with color and texture feature values more significant than one were retained for model building, respectively. This treatment can fully consider the contribution rate of each PC to improve the modeling accuracy and reduce the redundant information of the original features.

**Table 2:** Variance contribution rate of the principal

PC	Tillering stage				Elongation stage			
	CF		TF		CF		TF	
	E	CVCR	E	CVCR	E	CVCR	E	CVCR
1	14.427	61.726	3.319	79.941	12.204	50.002	3.988	78.437
2	5.465	85.106	0.546	93.091	8.409	84.456	0.731	92.804
3	2.249	94.729	0.244	98.967	2.513	94.750	0.351	99.716
4	0.483	96.795	0.041	99.944	0.566	97.071	0.013	99.966
5	0.314	98.137	0.002	100	0.368	98.578	0.002	100

PC: Principal Components; E: Eigenvalue; CVCR: Cumulative Variance Contribution Rate.

### Performance comparison of N content estimation models

Based on the analysis results, the image parameters with correlation coefficients greater than 0.7 in CA were selected to construct the strongly correlated term (SCT) prediction model, and the principal components with eigenvalues greater than 1 in the PCA results to construct the PCA prediction model. In the modelling process, the selected image parameters used color features (CF), texture features (TF) and color-texture integrated features (C-TIF) as independent variables and sugarcane N content as dependent variables to build the CF, TF and C-TIF based The N content of sugarcane leaves was predicted based on CF, TF and C-TIF.

#### Prediction model of N content at tillering stage

In the tillering stage, R, G, S, and V were selected for CF of the SCT model, and IDM, ASM, and ENT were selected for TF. The first three color PCs and the first texture PC were set for the PCA model. After validation, the optimal parameters for different models were selected. For the BPNN model, identity was chosen as the activation function, lbfgs was chosen as the weight optimizer, and the number of hidden layers and neurons in each hidden layer was set to 50. for the RF model, the optimal number of trees was set to 4, the minimum number of samples was 2, and the minimum number of pieces needed at the leaf nodes was 4. For the SFM, the number of principal components was retained at 4 for PLS; the SVR model kernel function is selected as rbf, and the kernel coefficients are determined as scale.

**Figure 3:**
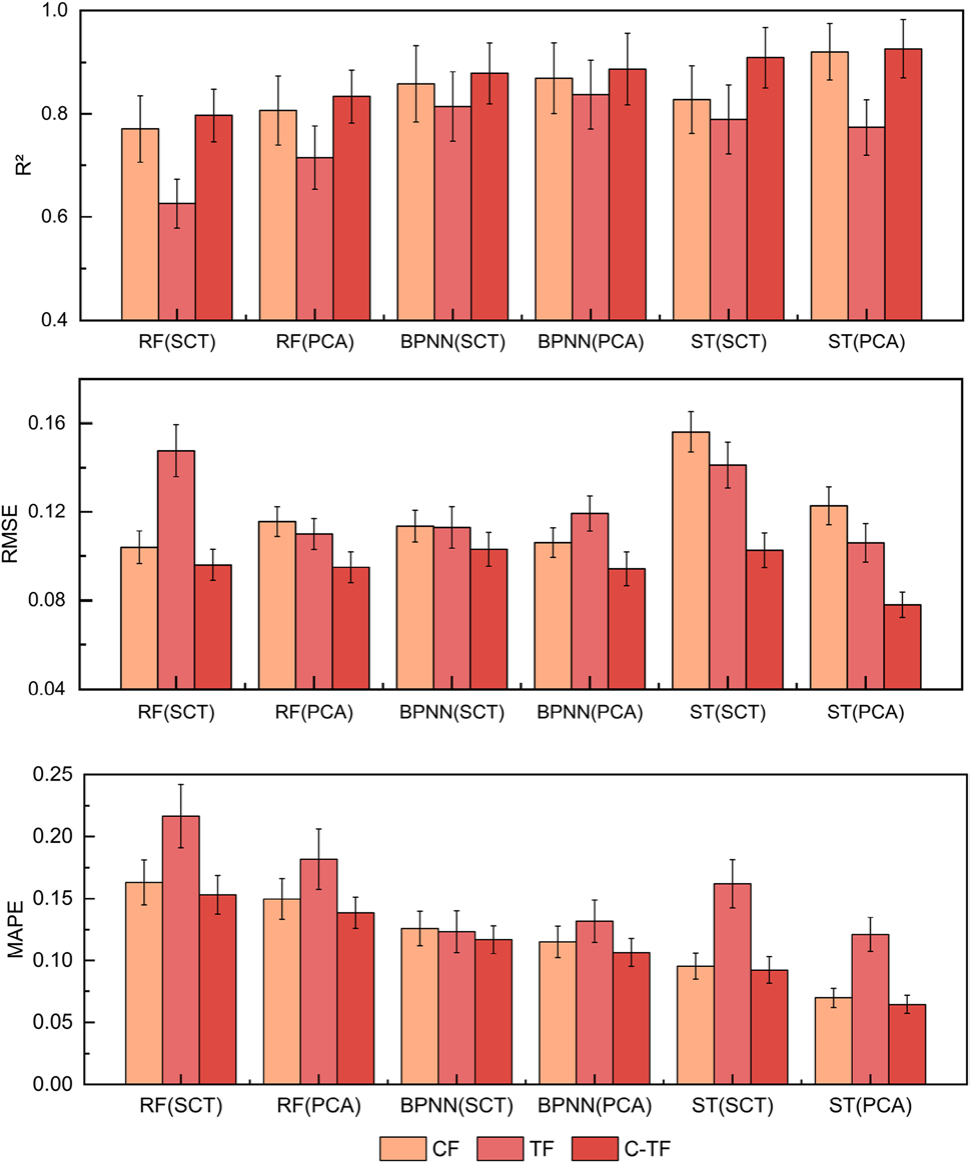
Comparison of model evaluation metrics based on different input variables at the tillering stage. Model performance was evaluated based on R^2^, RMSE and MAPE, and the performance of different models under different metrics was indicated using different colored bar graphs, respectively.

After obtaining the model training results, a comparison of the performance evaluation metrics of the same regression algorithm models with different input variables was carried out, as shown in Fig. 3. Among these models, the performance of the TF-based model was observed to be the worst, the CF-based model was the following best, and the version of the C-TIF-based model was considered to be the best. The R^2^ of the C-TIF model was recorded as a maximum improvement of 9.85% compared to the CF model, and both MAPE and RMSE were lower than the single feature model. With different characterization methods, it was found that the PCA model proved to have higher R^2^ than the SCT model, with a maximum enhancement of 5.37% and better performance of MAPE and RMSE. These results suggest that C-T-PCA as an input variable can train the most accurate model in sugarcane tillering.

**Figure 4:**
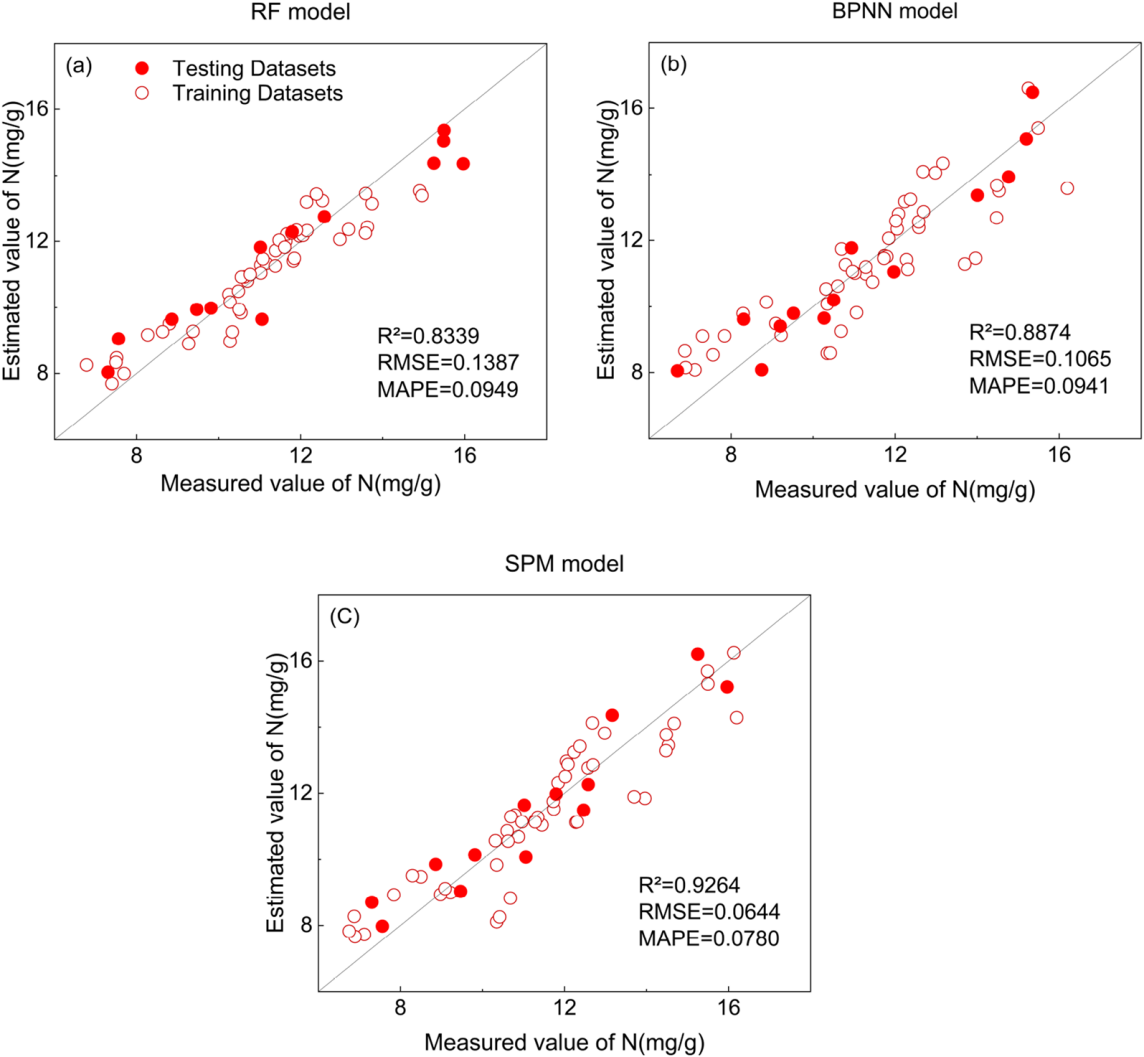
Scatter plot of RF, BPNN and SPM model prediction results based on C-T-PCA at the tillering stage. Model performance was evaluated based on R^2^, RMSE and MAPE.

The C-T-PCA features were used as input variables to compare the performance of different regression algorithms at the sugarcane tillering stage. The scatter plots of the prediction results are shown in Fig. 4. the model performance of RF was weaker than that of BPNN, SFM and other base models within SFM (PLS, SVR), and the R^2^ of the single-feature model fluctuated more. The model performance of BPNN compared with PLS and SVR showed a maximum improvement in R^2^ of 5.63% and 5.36%, respectively. The R^2^ of the SFM model is significantly higher than all the base models in its framework, with a maximum improvement of R^2^ of 5.42% and a significant decrease in both RMSE and MAPE. The SFM model based on C-T-PCA is the best model for N nutrient estimation in the sugarcane tillering stage.

#### Prediction model of N content at elongation stage

G, (G-R), H, and S were selected for the CF of the elongation stage SCT model. IDM, ASM, and ENT were selected for the TF. the first three color PCs, and the first texture PC was selected for the PCA model. the optimal parameters of the model were all the same as those of the tillering stage model.

**Figure 5:**
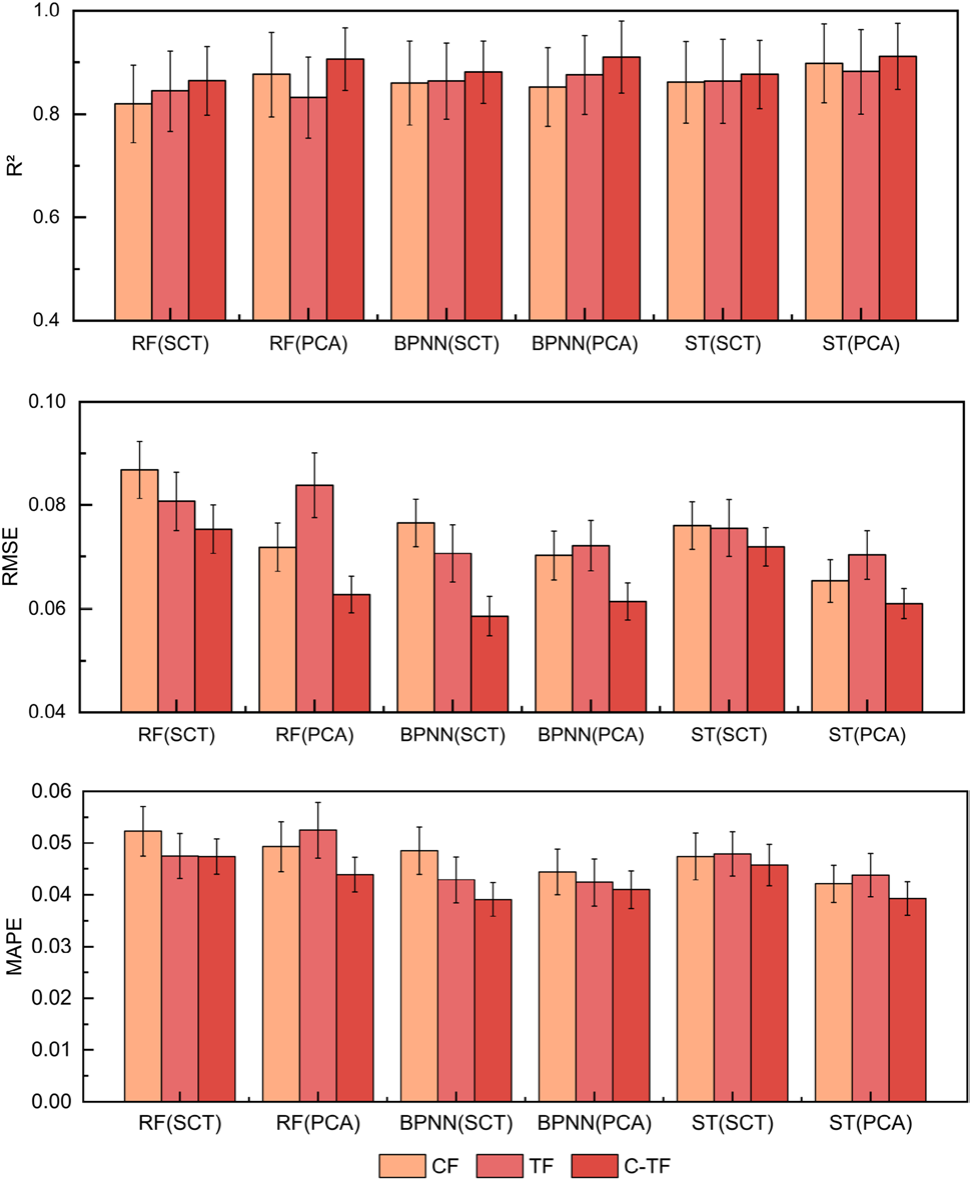
Comparison of model evaluation stage metrics based on different input variables in the elongation stage. Model performance is evaluated based on R^2^, RMSE and MAPE, and the performance of different models under different metrics is indicated using different colored bars, respectively.

After the model training results are obtained, the results are shown in Fig. 5 by comparing the effect of different input variables on the performance of the same regression algorithm model. There is no fixed size relationship for the R^2^ value in the single-feature models based on CF or TF, and its performance varies across different analytical methods and independent variables. In comparison, the R^2^ value of the C-TIF model performs better among all single-feature models with a maximum improvement of 8.91%, and the MAPE and RMSE values are also observed to be lower. When comparing the model-dependent variables of the different analytical approaches, the R^2^ values of the PCA model were found to be better than the SCT model in all cases, with a maximum enhancement of 4.81%. Also, the MAPE and RMSE values were lower than the SCT models. Thus, C-T-PCA was recognized as the best combination of input variables to use in the sugarcane elongation prediction model.

**Figure 6:**
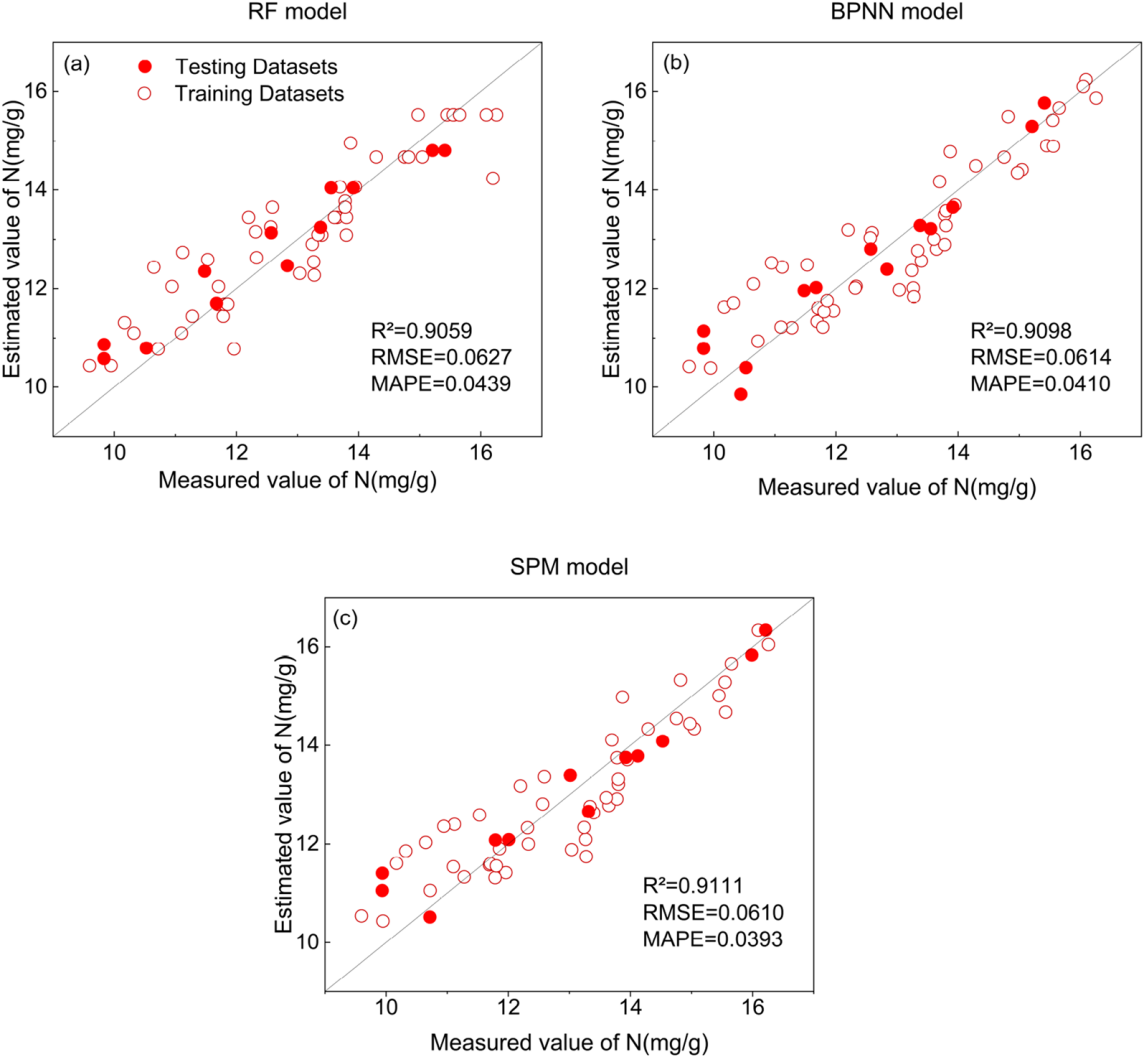
Scatter plot of the prediction results of the RF, BPNN and SPM models based on C-T-PCA for the elongation stage. Model performance was evaluated based on R^2^, RMSE and MAPE.

The model performance of different regression algorithms for sugarcane elongation was compared using C-T-PCA features as input variables. The scatter plot of the prediction results is shown in Fig. 6. The model performance of RF was observed to be weaker than that of BPNN and SFM but more robust than the other base models (PLS, SVR) within SFM. The model performance of BPNN was compared with PLS, SVR, and RF, and the maximum improvement in R^2^ was recorded as 4.98%, 8.30%, and 1.39%, respectively. Among all the models, the performance of SFM was the best, with a maximum enhancement of R^2^ of 8.46%, while RMSE and MAPE were also recorded as the lowest values. Thus, the SFM model based on C-T-PCA proved to be the best model for N nutrition estimation at the tillering stage of sugarcane.

## Discussion

### Estimation of crop N-nutrient nutrition using digital camera images of cane leaves

Digital camera images are widely used in machine vision because of their low cost, richly embedded information and easy extraction^56^. However, due to the environmental conditions and acquisition equipment, digital camera images are susceptible to uneven illumination and noise interference. This study used the MSRCR algorithm to de-illuminate the images to solve these problems. Also, it used the U-Net depth network structure to extract the leaf subjects, which removed most of the effecsts caused by environmental factors on the images. These treatments effectively improve the quality of feature extraction, and the images are directly used for quantitative analysis of crop nutrient status under natural lighting.

### Comparison of different regression models and model-independent variables

After model training and validation in sugarcane tillering and elongation periods, this study found that the model trained based on C-TIF performed best among the three feature types. In contrast, the model trained based on CF or TF single features performed differently in different growth periods. In addition, the accuracy of the models constructed from low-dimensional features obtained by PCA was higher than that of the models constructed using SCT features in both growth periods.

Among the three regression algorithms, SFM showed better stability and accuracy. The performance of the RF model varied widely in different growth periods, especially in the texture feature model at the tillering stage, where its performance fluctuated and lacked stability. This study used five-fold cross-validation to mitigate the effect of data set size on model performance evaluation and avoid model overfitting. The optimal model parameters were determined by grid search to improve the performance. SFM integrates multiple ML algorithms to reduce overfitting and autocorrelation of input variables and thus has strong generalization performance to maintain a highly accurate model for different growth periods and different input variables of sugarcane.

### Future research prospects

In this study, digital camera images were utilized in conjunction with machine learning techniques to estimate nitrogen nutrition parameters of sugarcane with high accuracy and stability, despite the relatively small sample size currently used. Currently, the diagnosis of crop nutrition is mainly based on the spectral information of images; however, images also contain a wealth of texture, structure, area, and relative spatial data, and these potential resources still need to be fully exploited and applied. Future research can use deep learning algorithms, such as convolutional neural networks and other models, for plant image feature extraction and recognition, including but not limited to texture, structure, area, and relative spatial features of the image, to improve the accurate recognition and quantitative analysis of crop nutritional status. Meanwhile, attempts can be made to use advanced deep learning models such as Generative Adversarial Networks (GAN) or Autoencoders to enhance or reconstruct the plant images to mine the potential information of the photos further. In addition, developing and using large-scale image datasets suitable for the agricultural field need to be considered to enhance the training efficiency and generalization ability of deep learning models. These deep-learning application directions and methods are expected to significantly improve crop nutrition diagnosis and quantitative analysis accuracy and efficiency. Diagnosis of N-vegetable nutrition is only the first step of precise N fertilizer management, and more research is needed to combine the diagnostic results with the correct N fertilizer application methods, which will further bring more significant optimization and benefits to agricultural production.

## Conclusions

This paper evaluated the effects of three machine learning regression models, three model-independent variables, and two feature analysis methods on the performance of sugarcane N substance nutrient parameter estimation. The results showed that the C-T-PCA-based SFM model performed the best in estimating N-nutrient parameters of sugarcane with R^2^ of 0.9264 and 0.9111 at the tillering and elongation stages, respectively. At the same time, the grid search and five-fold cross-validation methods were used to avoid model overfitting and improve model accuracy and generalization performance. According to the feature importance analysis, color features play a significant role in N-substance nutrition estimation, and adding texture features can somewhat improve the model prediction accuracy. Therefore, we believe combining digital images and appropriate machine learning algorithms can more quickly and reliably estimate the N-substance nutrient parameters of crops.

## Data Availability

The datasets generated during and/or analysed during the current study are available from the corresponding author on reasonable request.
